# Hippocampal lesions facilitate instrumental learning with delayed reinforcement but induce impulsive choice in rats

**DOI:** 10.1186/1471-2202-6-36

**Published:** 2005-05-13

**Authors:** Timothy HC Cheung, Rudolf N Cardinal

**Affiliations:** 1 Department of Experimental Psychology, University of Cambridge, Downing Street, Cambridge CB2 3EB, UK; 2 Psychopharmacology Section, Division of Psychiatry, B Floor, Medical School, Queen's Medical Centre, Nottingham NG7 2UH, UK

## Abstract

**Background:**

Animals must frequently act to influence the world even when the reinforcing outcomes of their actions are delayed. Learning with action-outcome delays is a complex problem, and little is known of the neural mechanisms that bridge such delays. When outcomes are delayed, they may be attributed to (or associated with) the action that caused them, or mistakenly attributed to other stimuli, such as the environmental context. Consequently, animals that are poor at forming context-outcome associations might learn action-outcome associations better with delayed reinforcement than normal animals. The hippocampus contributes to the representation of environmental context, being required for aspects of contextual conditioning. We therefore hypothesized that animals with hippocampal lesions would be better than normal animals at learning to act on the basis of delayed reinforcement. We tested the ability of hippocampal-lesioned rats to learn a free-operant instrumental response using delayed reinforcement, and what is potentially a related ability – the ability to exhibit self-controlled choice, or to sacrifice an immediate, small reward in order to obtain a delayed but larger reward.

**Results:**

Rats with sham or excitotoxic hippocampal lesions acquired an instrumental response with different delays (0, 10, or 20 s) between the response and reinforcer delivery. These delays retarded learning in normal rats. Hippocampal-lesioned rats responded slightly less than sham-operated controls in the absence of delays, but they became better at learning (relative to shams) as the delays increased; delays impaired learning less in hippocampal-lesioned rats than in shams. In contrast, lesioned rats exhibited impulsive choice, preferring an immediate, small reward to a delayed, larger reward, even though they preferred the large reward when it was not delayed.

**Conclusion:**

These results support the view that the hippocampus hinders action-outcome learning with delayed outcomes, perhaps because it promotes the formation of context-outcome associations instead. However, although lesioned rats were better at learning with delayed reinforcement, they were worse at choosing it, suggesting that self-controlled choice and learning with delayed reinforcement tax different psychological processes.

## Background

When one event or stimulus in the world reliably precedes and predicts another, animals readily learn the predictive relationship, exemplified by Pavlovian conditioning. Similarly, when an animal's own actions cause (and thus predict) some outcome, animals learn this relationship (an aspect of instrumental or operant conditioning). Frequently, however, antecedent and consequent events are separated in time. When animals act to obtain reinforcement, the final outcomes do not always follow the actions immediately; thus, animals must learn instrumental action-outcome contingencies using delayed reinforcement. Delays can hamper both Pavlovian and instrumental conditioning [1-5]: for example, although animals can bridge substantial delays to acquire instrumental responses, instrumental conditioning has long been observed to be systematically impaired as the outcome is delayed [6-11]. Furthermore, individual variation in the ability to use delayed reinforcement may determine one aspect of impulsivity: an animal able to forgo short-term poor rewards in order to obtain delayed but better rewards may be termed self-controlled, whereas an animal that cannot tolerate delays to reward may be said to exhibit impulsive choice [12-15].

There are several psychological reasons why action-outcome delays might impair learning or performance of an instrumental response [12,16]. Instrumental responding is controlled by several processes [4,17,18]; for example, rats work for outcomes that they value, using knowledge of the action-outcome contingencies in force to produce goal-directed actions. They also develop direct stimulus-response (S-R) associations, or habits. Action-outcome delays might, therefore, reduce the instrumental incentive value of the goal: valuing the goal less, animals may work less for it. Similarly, delays may hinder animals' ability to perceive the action-outcome contingency. Delayed rewards may also be less effective at reinforcing S-R habits. It is presently not known whether responses acquired with delayed reinforcement are governed by a different balance of habits and goal-directed actions than responses acquired with immediate reinforcement. However, one important factor in learning to act using delayed reinforcement may be the role of the environmental context. The animal's task is to attribute the outcome to its actions; instead, it may erroneously associate the outcome with the context, since the context is a cue that is temporally closer to the outcome than the action. The longer the delay, the more this contextual competition comes to impair the learning of the action-outcome contingency. Instrumental conditioning with delayed reinforcement can be enhanced if rats are exposed to the relevant contextual cues prior to instrumental training, and this enhancement is lessened if 'free' (non-contingent) rewards are given during the contextual pre-exposure periods [9,17]. These results are consistent with the theory that during the action-outcome delay, contextual cues compete with the action to become associated with the outcome; pre-exposing the animals to the context with no consequences reduces this contextual competition, by making the context a bad predictor of the outcome (perhaps via latent inhibition or learned irrelevance), and this in turn makes the action-outcome contingency more salient and easier to learn [9,17].

Little is known of the neural basis of instrumental learning with delayed reinforcement [16]. However, there is good evidence that the hippocampus contributes to the representation of context. Lesions of the hippocampal formation (H) have been shown to impair Pavlovian conditioning to a contextual conditioned stimulus (CS), but not to a discrete CS, in rats [19-31], at least for some processes involving contextual representation [32-34]. Since context-outcome associations are thought to hinder instrumental learning with delayed reinforcement (contextual competition) [9,17], it follows that if H lesions impair the formation of associations involving the context, such lesions might reduce contextual competition and hence *facilitate *instrumental conditioning when there is an action-outcome delay.

**Figure 1:**
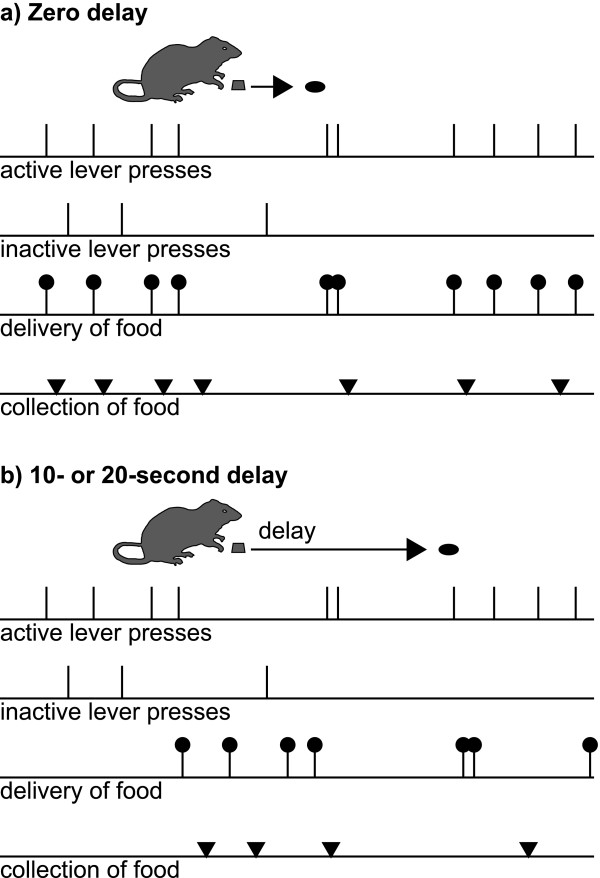
**Task schematic: free-operant instrumental responding on a fixed-ratio-1 (FR-1) schedule with delayed reinforcement. **Subjects are offered two levers; one (the active lever) delivers a single food pellet for every press (an FR-1 schedule) and the other (the inactive lever) has no programmed consequence. Food can either be delivered immediately **(a) **or after a delay **(b) **following responses on the active lever. The levers remain available throughout the session (hence, free-operant responding). Events of interest are lever presses, delivery of food pellets, and collection of food by the rat (when it pokes its nose into the food alcove following food delivery). To obtain food, the hungry rat must discriminate the active from the inactive lever, which is more difficult when the outcome is delayed. In these examples, the rat's response patterns (active and inactive lever presses, and collection of food) are fictional, while food delivery is contingent upon active lever pressing.

**Figure 2:**
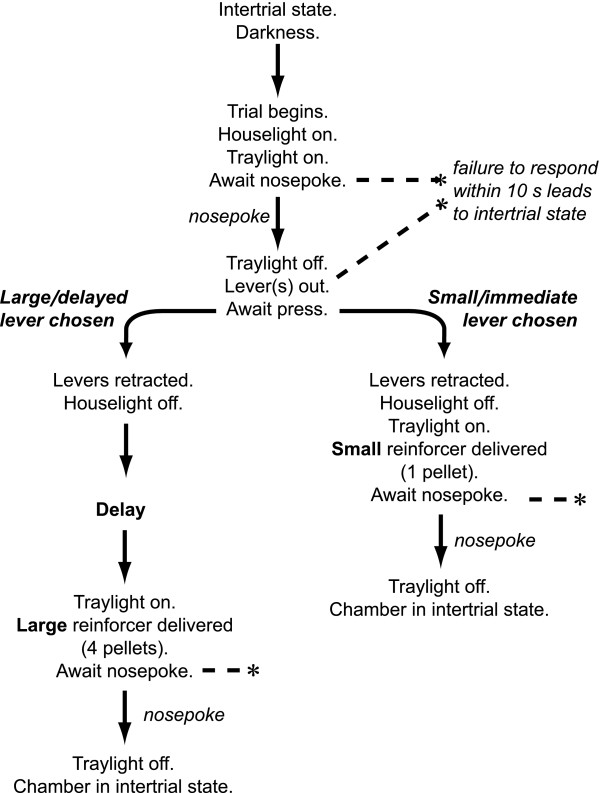
**Task schematic: choice between small, immediate and large, delayed reward. **Delayed reinforcement choice task [35, 86, 99], based on that of Evenden and Ryan [107]. Hungry rats regularly choose between two levers. Responding on one lever leads to the immediate delivery of a small food reward (1 pellet); responding on the other leads to a much larger food reward (4 pellets), but this reward is delayed for between 0 and 60 seconds. The figure shows the format of a single trial; trials begin at regular intervals (every 100 s), so choice of the small reinforcer is always suboptimal. Sessions consist of 5 blocks. In each block, two single-lever trials are given (one trial for each lever, in random order), to ensure the animals sample the options available at that time; these are followed by ten choice trials. The delay to the large reinforcer is varied systematically across the session: delays for each block are 0, 10, 20, 40, and 60 s respectively.

To investigate whether the hippocampus contributes to learning with delayed reinforcement, we examined the ability of rats with excitotoxic lesions of the hippocampus to acquire instrumental responding with delayed reward, comparing them to sham-operated controls. Each subject was allowed to respond freely on two levers, one of which produced reinforcement after a delay of 0, 10, or 20 s (Figure 1). We report that H-lesioned rats were slightly impaired at learning the lever-press response in the absence of delays. Delays retarded learning in sham-operated controls, but the delays did not impair the H-lesioned rats to the same extent. Thus, as the delays were increased, H-lesioned rats became better at learning relative to controls, suggesting that the presence of delays had less of an effect on H-lesioned rats. To establish whether this relative improvement in learning with delayed reinforcement would also manifest itself as improved self-control, we also trained a different group of rats on a task in which they had to choose between an immediate, small reward and a delayed, large reward (Figure 2) and made excitotoxic hippocampal lesions before retesting the rats postoperatively. Good learning with delayed reinforcement did not translate to self-controlled choice. We report that H lesions severely impaired rats' ability to choose the larger reward when it was delayed, but not when the delay preceding delivery of the large reward was removed, demonstrating that hippocampal lesions induce impulsive choice.

## Results

### Histology

In Experiment 1, there were five postoperative deaths. No rats were excluded after histological analysis; final group sizes were 9 (H, 0 s delay), 5 (sham, 0 s delay), 9 (H, 10 s delay), 6 (sham, 10 s delay), 9 (H, 20 s delay), and 5 (sham, 20 s delay). In Experiment 2, there was one postoperative death (H group), and one rat (sham group) fell ill five sessions after surgery and was killed. Histological analysis revealed that the lesions were incomplete or encroached significantly on neighbouring structures in 3 subjects. These subjects were excluded; final group sizes were therefore 7 (sham) and 12 (H).

**Figure 3:**
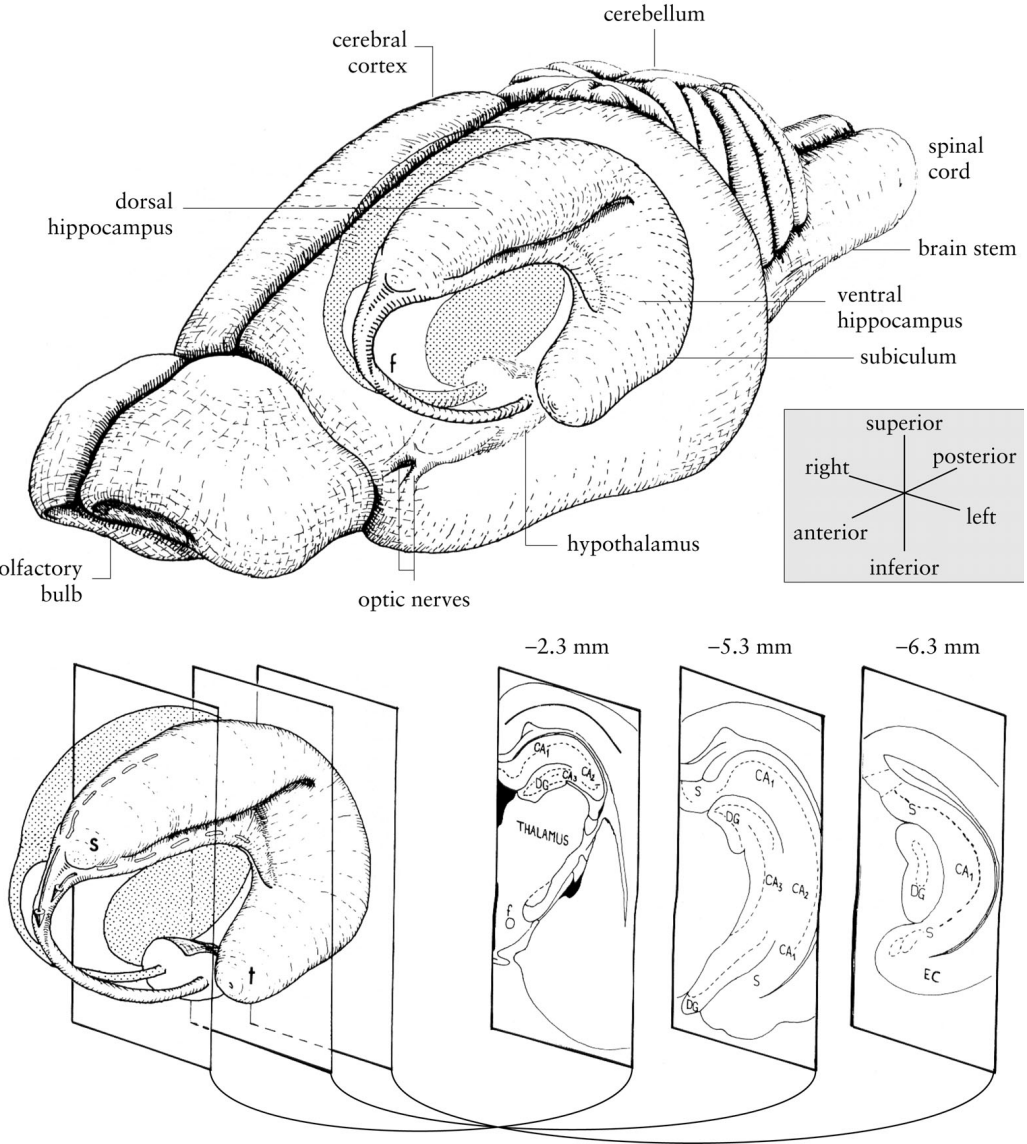
**Diagram of the rat hippocampus. **Drawings of the rat brain showing the three-dimensional organization of the hippocampus and related structures. Three coronal sections through the left hippocampus are shown at the bottom right of the figure, with their approximate anteroposterior coordinate relative to bregma. CA1, CA2, CA3: cornu ammonis fields 1–3; DG: dentate gyrus; EC: entorhinal cortex; f: fornix; s: septal pole of the hippocampus; S: subiculum; t: temporal pole of the hippocampus. Adapted from Figure 1 of ref. [113], copyright (1995), with permission from Elsevier.

**Figure 4:**
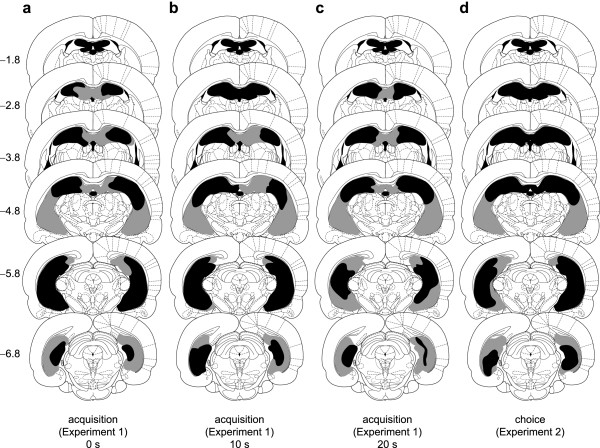
**Schematic of lesions of the hippocampus. **Black shading indicates the extent of neuronal loss common to all subjects (and also the third and lateral ventricles); grey indicates the area lesioned in at least one subject. Coronal sections are (from top to bottom) -1.8, -2.8, -3.8, -4.8, -5.8, and -6.8 mm relative to bregma. Diagrams are modified from ref. [114]. Panels **a-c **show schematics for Experiment 1 (acquisition of a free-operant instrumental response with delayed reinforcement; 0 s, 10 s, and 20 s groups, respectively) while **d **shows schematics for Experiment 2 (choice between small, immediate and large, delayed reinforcement).

**Figure 5:**
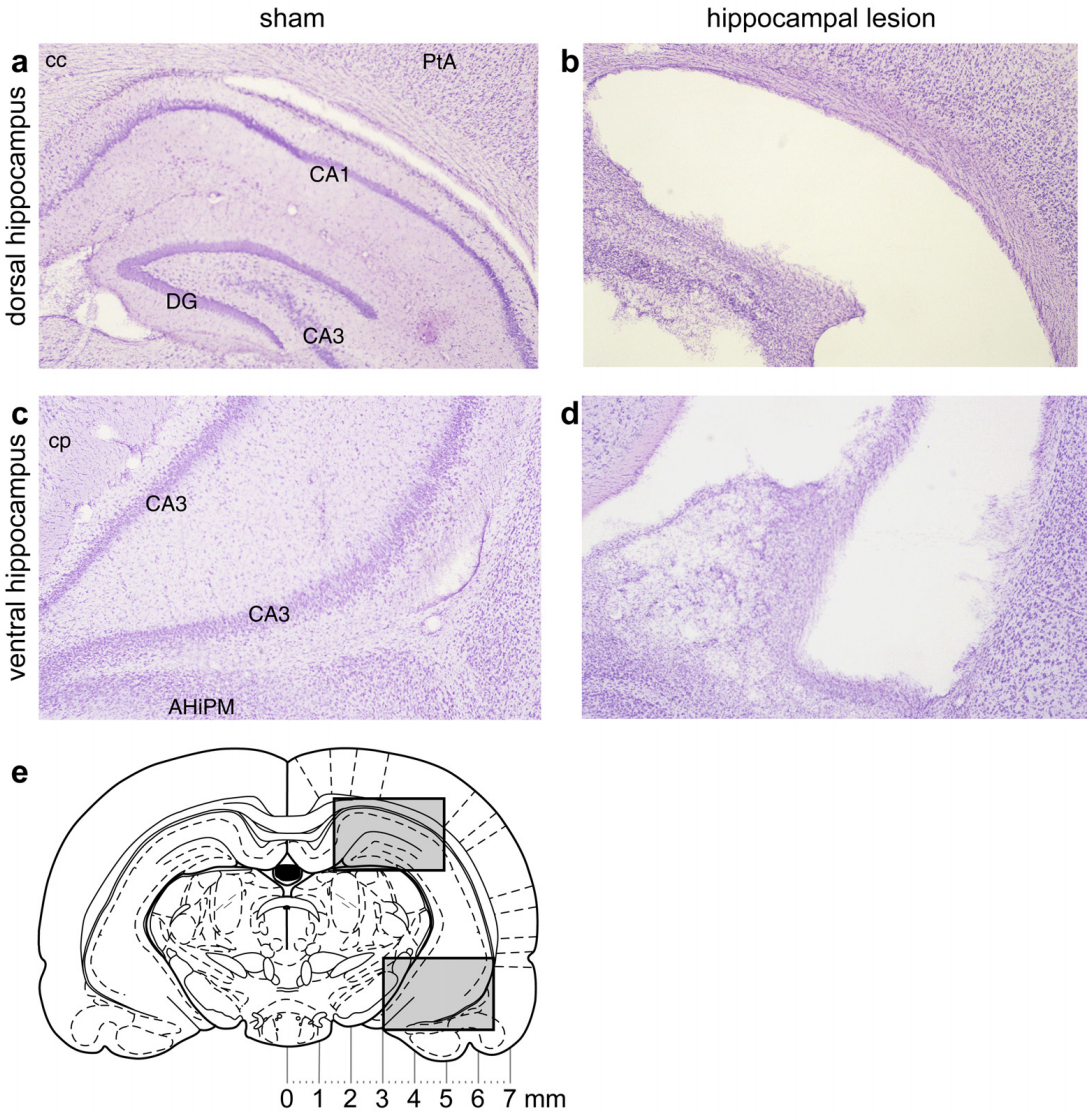
**Photomicrographs of lesions of the hippocampus. **Lesions of the hippocampus: photomicrographs of sections ~4.7 mm posterior to bregma, stained with cresyl violet. **(a) **Sham-operated rat, dorsal hippocampus, right hemisphere (medial to the left). CA1, cornu ammonis field 1; CA3, cornu ammonis field 3; DG, dentate gyrus; cc, corpus callosum; PtA, parietal association cortex. **(b) **Hippocampal-lesioned rat; same area as (a). There is tissue collapse within the lesion and the ventricle is greatly expanded. **(c) **Sham-operated rat, ventral hippocampus. AHiPM, amygdalohippocampal area, posteromedial part; cp, cerebral peduncle. **(d) **Hippocampal-lesioned rat, same area as (c). **(e) **Coronal diagram of the rat brain at 4.8 mm posterior to bregma [114], with scale. The upper grey box indicates approximately the region shown in (a) and (b); the lower grey box indicates approximately the region shown in (c) and (d).

A diagram of the rat hippocampus is shown in Figure 3. Lesions of the hippocampus encompassed much of the dorsal and ventral hippocampal pyramidal cell (cornu ammonis CA1-CA3) fields, the dentate gyrus, the subiculum, and the fimbriae. Neuronal loss and associated gliosis extended in an anteroposterior direction from approximately -0.8 mm to -7.8 mm relative to bregma (negative coordinates are posterior). Damage to the dorsal and ventral hippocampal commissure was occasionally seen, but damage to the overlying cortex was minimal. Schematics of the lesions are shown in Figure 4, and photomicrographs of a representative lesion are shown in Figure 5.

### Acquisition of instrumental responding (experiment 1)

**Figure 6:**
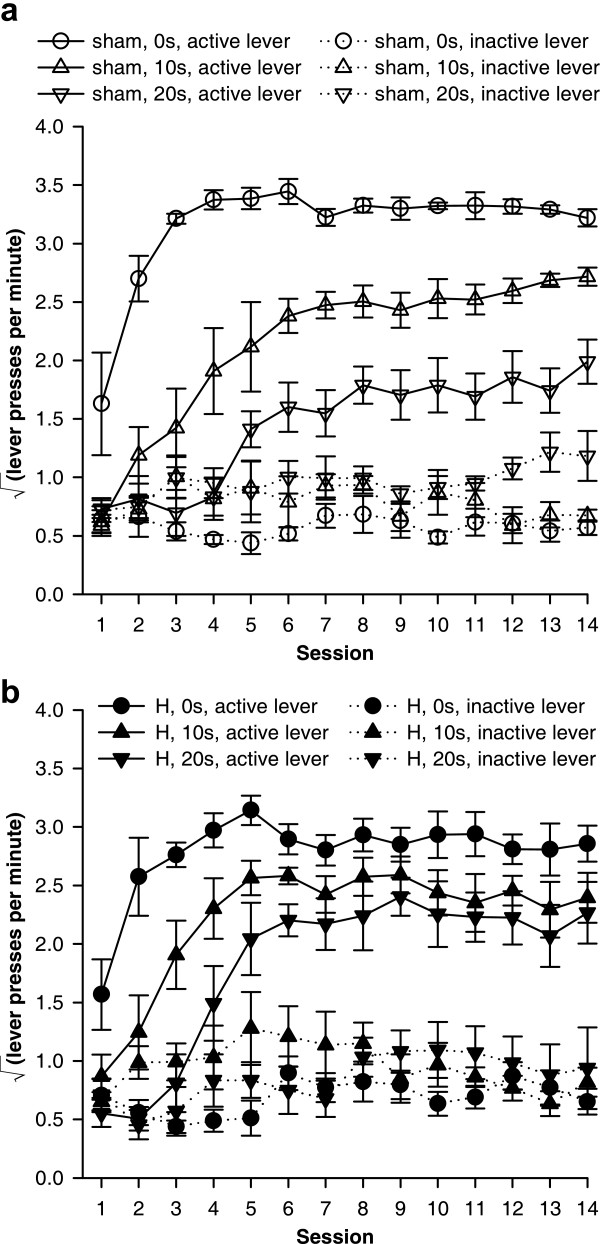
**Effects of delays to reinforcement on acquisition of free-operant responding under an FR-1 schedule. **Data plotted to show the effects of delays. All groups discriminated between the active and the inactive lever, and delays retarded acquisition of the active lever response in both groups. **(a) **Responding of sham-operated control rats, under all three response-reinforcer delay conditions. **(b) **Responding of hippocampal-lesioned rats under all delay conditions. The next figure replots these data to show the effect of the lesion more clearly.

**Figure 7:**
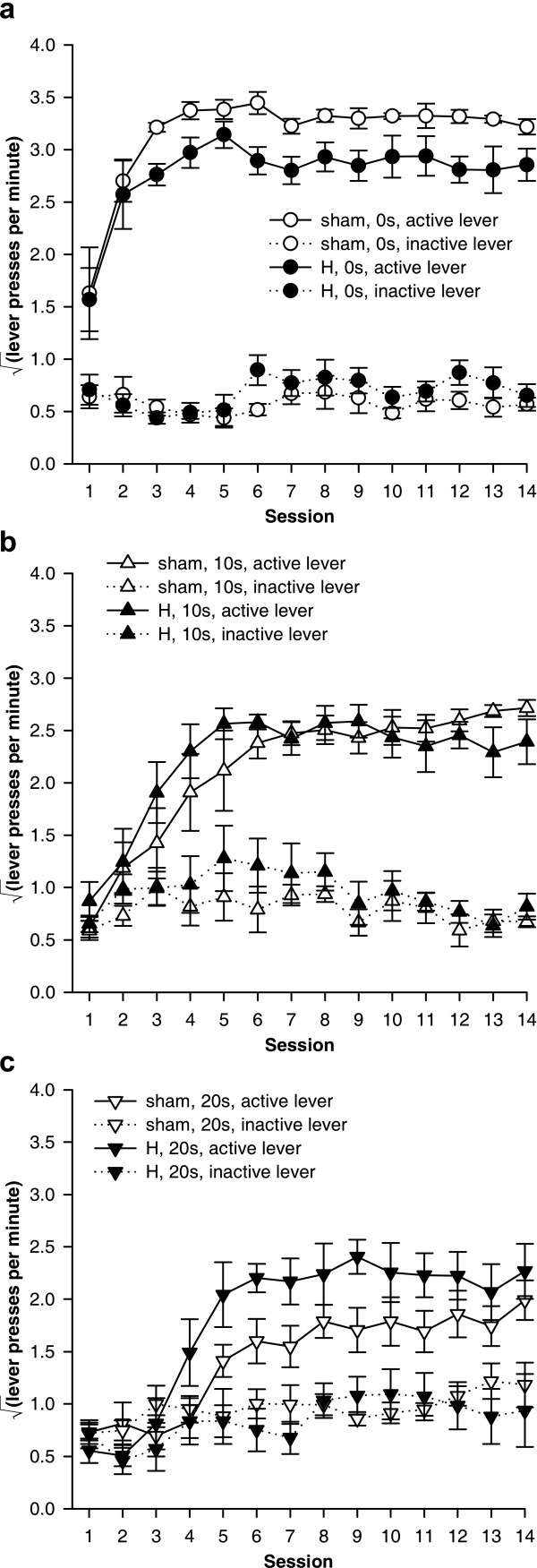
**Effect of hippocampal lesions on acquisition of free-operant responding with delayed reinforcement. **Data plotted to show the effects of hippocampal lesions (same data as in the previous figure). There was a delay-dependent impairment in H-lesioned rats (significant lesion × delay interaction, see text), who learned less well than shams only when reinforcement was *not *delayed. **(a) **With a delay of 0 s, H-lesioned rats responded less on the active lever than shams did. **(b) **With a 10 s delay, H-lesioned rats responded the same as shams. **(c) **With a 20 s delay, H-lesioned rats responded more than shams on the active lever, though this difference was not statistically significant on its own.

As expected, response-reinforcer delays retarded the acquisition of instrumental responding in sham-operated rats (Figure 6a). However, this impairment was lessened in H-lesioned rats (Figure 6b). H-lesioned rats responded less than shams in the absence of a response-reinforcer delay (Figure 7a), but responded as well as shams when delays were imposed (Figure 7b,c); H-lesioned rats were even facilitated numerically relative to shams in the 20 s delay condition (Figure 7c), though this difference was not statistically significant on its own. These conclusions were reached statistically as follows.

An overall ANOVA, using the model lesion_2 _× delay_3 _× (session_14 _× lever_2 _× S), revealed a lesion × lever × delay interaction (*F*_2,37 _= 4.16, *p *= .023), justifying sub-analyses, in addition to effects of delay (*F*_2,37 _= 17.9, *p *< .001), lever (*F*_1,37 _= 435, *p *< .001), delay × lever (*F*_2,37 _= 4.16, *p *< .001), session (*F*_5.35,198.0 _= 38.7,  = .412, *p *< .001), delay × session (*F*_10.7,198.0 _= 3.03, *p *= .001), session × lever (*F*_4.99,184.6 _= 17.5,  = .384, *p *< .001), and delay × session × lever (*F*_10.0,184.6 _= 2.30,  = .384, *p *= .015). The differences between the groups were in their responding on the active lever (active lever, lesion × delay: *F*_2,37 _= 3.71, *p *= .034) rather than on the inactive lever (inactive lever, terms involving lesion: maximum *F*_2,37 _= 1.146, NS). All six groups learned to discriminate between the two levers, responding more on the active lever than on the inactive lever (*p *< .05, main effect of lever for each group).

Delays reduced the rate of acquisition and the final level of responding on the active lever for sham-operated rats (Figure 6a; delay, *F*_2,13 _= 58.7, *p *< .001; delay × session, *F*_10.8,70.4 _= 2.67,  = .417, *p *= .007). Delays also increased responding on the inactive lever somewhat (Figure 6a; delay: *F*_2,13 _= 5.26, *p *= .021; delay × session, *F*_13.1,85.2 _= 1.22,  = .504, NS). Similarly, in H-lesioned rats, delays reduced responding on the active lever (Figure 6b; delay: *F*_2,24 _= 12.3, *p *< .001; delay × session: *F*_7.8,93.1 _= 2.76,  = .298, *p *= .009), although they did not significantly affect responding on the inactive lever (delay: *F*_2,24 _= 1.91, NS; delay × session: *F*_12.3,147.9 _= 1.37,  = .474, NS).

At 0 s delay, H-lesioned rats responded significantly less than shams on the active lever (Figure 7a; lesion: *F*_1,12 _= 6.11, *p *= .029). There were no differences in responding on the inactive lever (*F*s < 1, NS). At 10 s delay, there were no differences between sham-operated and H-lesioned rats in responding on either the active or the inactive lever (*F*s < 1.35, *p *≥ .266). At 20 s delay, there were also no significant differences on either lever (active lever: lesion *F*_1,12 _= 2.485, *p *= .141, lesion × session *F *< 1, NS; inactive lever: *F*s < 1, NS), although the H-lesioned rats responded numerically more than shams on the active lever throughout.

Inspection of Figure 6 also suggested that delays had less of an impact on the final (asymptotic) rates of responding in H-lesioned rats than in shams. The sessions were divided by eye into an acquisition phase (sessions 1–6) and a 'stable' phase (sessions 7–14). Responding on the active lever in the 'stable' phase was analysed; this revealed a lesion × delay interaction (*F*_2,37 _= 3.44, *p *= .043), with delays markedly reducing stable rates of responding in shams (*F*_2,13 _= 42.3, *p *< .001) but less so in H-lesioned rats (*F*_2,24 _= 3.11, *p *= .063).

### Experienced response-delivery and response-collection delays (experiment 1)

For every reinforcer delivered, the active lever response most closely preceding it in time was identified, and the time between that response and delivery of the reinforcer (the 'response-delivery delay') was calculated. This time can therefore be equal to or less than the programmed delay, and is only relevant for subjects experiencing non-zero programmed response-reinforcer delays. The response-to-reinforcer-collection ('response-collection') delays were also calculated: for every reinforcer delivered, the response most closely preceding it and the nosepoke most closely following it were identified, and the time between these two events calculated. This time can be shorter or longer than the programmed delay, and is relevant for all subjects.

**Figure 8:**
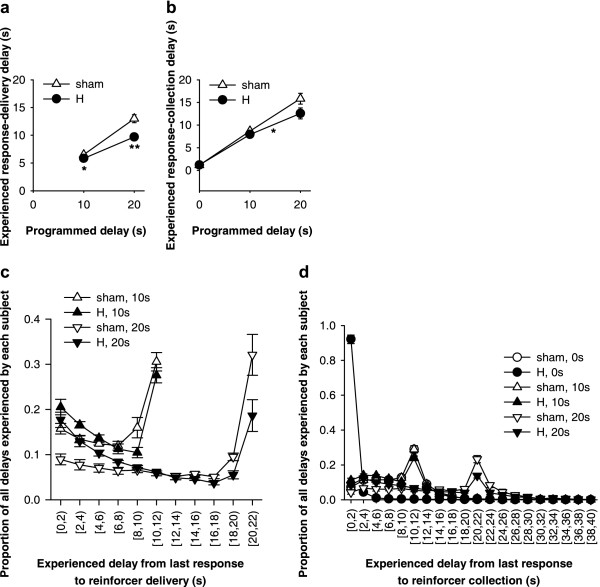
**Programmed and experienced delays to reinforcement. **H-lesioned rats experienced slightly shorter response-delivery delays (the delay between the most recent lever press and pellet delivery) than shams, and slightly shorter response-collection delays (the delay between the most recent lever press and pellet collection). **(a) **Mean experienced response-delivery delays (one value calculated per subject). When the programmed delay was 0 s, reinforcers were delivered immediately so no data are shown. H-lesioned rats experienced shorter response-delivery delays when the programmed delay was 10 s (* *p *= .012) or 20 s (** *p *= .009). **(b) **Mean experienced response-collection delays (one value calculated per subject). H-lesioned rats experienced slightly shorter delays overall (* *p *= .047, main effect of lesion), but the experienced delays did not differ significantly at any given programmed delay. **(c) **Distribution of experienced response-delivery delays. All experienced delays for a given subject were aggregated across all sessions, and the proportion falling into different 2-s ranges were calculated to give one value per range per subject; the graphs show means ± SEMs of these values. The interval notation '[*a*, *b*)' indicates that a given delay *x *falls in the range *a* ≤ *x* <*b*. H-lesioned rats experienced slightly fewer long delays and slightly more short delays in the 10 s condition (*p *= .019) and in the 20 s condition (*p *= .014). **(d) **Distribution of experienced response-collection delays, displayed in the same manner as (c). There were no differences in the distribution of delays experienced by H-lesioned and sham rats in the 0 s condition. In the 10 s condition, H-lesioned rats experienced a slightly lower proportion of long delays and a slightly higher proportion of short delays (*p *= .009), and similarly in the 20 s condition (*p *= .001).

H-lesioned rats experienced slightly shorter response-delivery delays than shams when the programmed delay was 10 s or 20 s (Figure 8a): there was a lesion × programmed delay interaction (*F*_1,25 _= 6.28, *p *= .019), and simple effects of the lesion when the programmed delay was 10 s (*F*_1,13 _= 8.49, *p *= .012) and when it was 20 s (*F*_1,12 _= 9.50, *p *= .009).

H-lesioned rats also experienced slightly shorter response-collection delays across all programmed delays (Figure 8b) (lesion: *F*_1,37 _= 4.21, *p *= .047), though the difference was not significant at any one programmed delay (lesion × programmed delay: *F*_2,37 _= 2.35, *p *= .109; simple effects of the lesion at different programmed delays: maximum *F*_1,12 _= 3.08, *p *= .105).

These differences in the *mean *delay experienced by each rat were reflected in differences in the *distribution *of response-delivery and response-collection delays when the programmed delay was non-zero (Figure 8c,d). All experienced delays for a given subject were aggregated across all sessions, and the proportion falling into different 2-s ranges were calculated to give one value per range per subject. For response-delivery delays, H-lesioned rats experienced slightly fewer long delays and slightly more short delays in the 10 s condition (lesion × range, *F*_3.6,47.0 _= 3.40,  = .723, *p *= .019) and in the 20 s condition (lesion × range, *F*_1.4,16.6 _= 6.54,  = .138, *p *= .014). For response-collection delays, there were no differences in the distribution of delays experienced by H-lesioned and sham rats in the 0 s condition (lesion and lesion × range, *F*s < 1, NS). In the 10 s condition, H-lesioned rats experienced a slightly lower proportion of long response-collection delays and a slightly higher proportion of short response-collection delays (lesion × range, *F*_4.4,57.6 _= 3.60,  = .233, *p *= .009). Similarly, in the 20 s condition, H-lesioned rats experienced a slightly lower proportion of long response-collection delays and a slightly higher proportion of short response-collection delays than shams (lesion × range, *F*_3.1,37.5 _= 7.02,  = .164, *p *= .001).

Since H-lesioned rats experienced slightly shorter delays than sham-operated rats, it was necessary to take this into account when establishing the effect of delays on learning, as follows.

### Effect of delays on learning (experiment 1)

**Figure 9:**
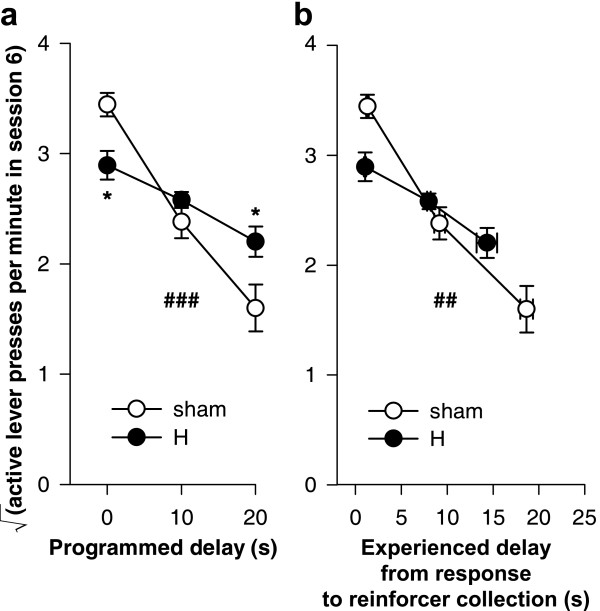
**Learning as a function of programmed and experienced delays to reinforcement. **The imposition of response-reinforcer delays systematically retarded the acquisition of free-operant instrumental responding, but this effect was lessened in H-lesioned rats, even allowing for differences in experienced response-collection delays. **(a) **The rate of lever-pressing in session 6 is plotted against the programmed response-reinforcer delay. There was a lesion × delay interaction (### *p *= .001): H-lesioned rats responded less than shams at zero delay (* *p *= .015), were not significantly different from shams at 10 s delay (*p *= .197), and responded more than shams at 20 s (* *p *= .028). **(b) **Responding in session 6 plotted against the experienced response-to-reinforcer collection delays for sessions 1–6 (vertical error bars: SEM of the square-root-transformed number of responses in session 6; horizontal error bars: SEM of the experienced response-collection delay, calculated up to and including that session). The gradients of the two lines differed significantly (## *p *= .002; see text), indicating that the relationship between experienced delays and responding was altered in H-lesioned rats.

There was a systematic relationship between the acquisition rate and the programmed delay of reinforcement, and this was altered in H-lesioned rats, who were less impaired by delays (compared to their performance at zero delay) than shams were. Figure 9a replots the rates of lever-pressing on session 6, at the end of the initial 'acquisition' phase. Despite the comparatively low power of such an analysis, lever-pressing was analysed for this session only, using the model lesion_2 _× delay_3 _× S. This revealed a significant lesion × delay interaction (*F*_2,37 _= 8.67, *p *= .001), which was analysed further. Increasing delays significantly reduced the rate of responding in this session for shams (*F*_2,13 _= 31.4, *p *< .001) and H-lesioned rats (*F*_2,24 _= 8.88, *p *= .001). H-lesioned rats responded less than shams at zero delay (*F*_1,12 _= 8.08, *p *= .015), were not significantly different from shams at 10 s delay (*F*_1,13 _= 1.848, *p *= .197), and responded more than shams at 20 s delay (*F*_1,12 _= 6.23, *p *= .028).

Since the H group experienced slightly shorter response-delivery and response-collection delays than shams when the programmed delay was non-zero (Figure 8), it is important to establish whether this effect alone was responsible for the lesser effect of delays on learning, or whether the effect of delays on H-lesioned rats was lessened over and above any effect to decrease the experienced delay. The mean experienced response-collection delay was calculated for each subject up to and including session 6. The square-root-transformed number of lever-presses in session 6 was then analysed using a general linear model of the form lesion_2 _× experienced delay_cov _× S; unlike a standard analysis of covariance, the factor × covariate interaction term was included in the model. This confirmed that the detrimental effects of delay upon learning were reduced in H-lesioned rats, compared to controls, over and above the differences in experienced delay (Figure 9b; lesion × experienced delay: *F*_1,39 _= 10.8, *p *= .002).

### Experienced delays and learning on the inactive lever (experiment 1)

No such delay-dependent lesion effects were observed for the inactive lever. Experienced inactive-response-delivery delays (calculated across all sessions in the same manner as for the active lever) were much longer and more variable than corresponding delays for the active lever, because subjects responded on the inactive lever so little. Means ± SEMs were 271 ± 31 s (sham, 0 s), 241 ± 23 s (H, 0 s), 201 ± 45 s (sham, 10 s), 184 ± 45 s (H, 10 s), 127 ± 21 s (sham, 20 s), and 171 ± 36 s (H, 20 s). ANOVA of these data showed that these experienced inactive-response-delivery delays depended upon the programmed active-response-delivery delay (delay: *F*_2,37 _= 3.80, *p *= .032) but there was no effect of the lesion and no lesion × delay interaction (*F*s < 1, NS). Experienced inactive-response-collection delays were 272 ± 31 s (sham, 0 s), 242 ± 23 s (H, 0 s), 204 ± 45 s (sham, 10 s), 186 ± 45 s (H, 10 s), 130 ± 21 s (sham, 20 s), and 174 ± 35 s (H, 20 s). Again, ANOVA that these experienced delays depended upon the programmed active-response-delivery delays (delay: *F*_2,37 _= 3.68, *p *= .035) but there was no effect of the lesion and no lesion × delay interaction (*F*s < 1, NS). When the square-root-transformed number of responses on the inactive lever in session 6 was analysed with the experienced delays up to that point as a predictor, using the model lesion_2 _× experienced inactive-response-collection delay_cov _× S just as for the active lever analysis, there was no lesion × experienced delay interaction; neither was there an effect of lesion or experienced delay (maximum *F*_2,37 _= 1.54, NS).

### Choice between an immediate, small reward and a large, delayed reward (experiment 2)

**Figure 10:**
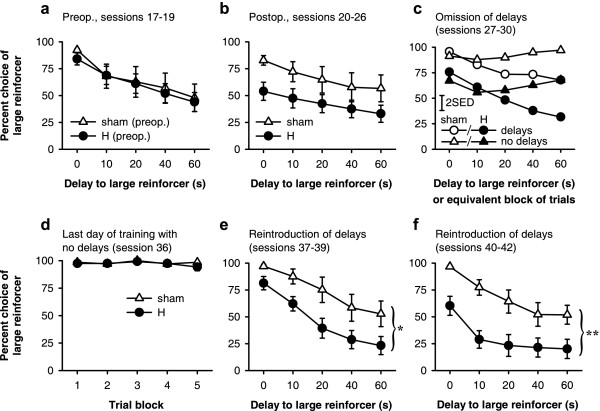
**Effects of hippocampal lesions on choice between immediate, small reward and large, delayed reward. (a) **Pattern of choice in the last three sessions before surgery; the sham and lesion groups were matched for performance. Rats' preference for the large reinforcer declined with delay (*p *< .001). **(b) **Choice in the first seven postoperative sessions. Although there was a change in behaviour in the lesioned group (lesion × pre/postop., *p *= .021), the difference between the two groups was not significant in its own right for these sessions (*p *= .08). **(c) **Effects of omitting all delays in alternating sessions (error bar, 2 SED for the three-way interaction). H-lesioned rats remained sensitive to the contingencies, altering their behaviour in response to delay omission, as shams did. **(d) **Last of six further consecutive sessions in which delays were omitted. Both groups preferred the large reinforcer strongly when it was not delayed, with no differences between sham and H-lesioned rats. **(e) **First three sessions following reintroduction of delays. Hippocampal-lesioned rats were impulsive, choosing the large, delayed reinforcer less often than shams (* *p *= .027). **(f) **Next three sessions following reintroduction of delays. Hippocampal-lesioned rats remained impulsive (** *p *= .007), and generalization between trial blocks occurred, reducing their preference for the large reinforcer in the zero-delay block as well (see text).

Preoperatively, subjects preferred the larger reinforcer less when it was delayed, and the groups remained matched following later histological selection (Figure 10a). Choice ratios (percent choice of the large, delayed reinforcer, calculated for all free-choice trials on which subjects responded) from the last 3 preoperative sessions were analysed using the model lesion intent_2 _× (delay_5 _× S). While there was a strong effect of delay (*F*_2.2,36.9 _= 22.9, *p *< .001), no terms involving lesion intent were significant (*F*s < 1, NS).

The choice patterns of the two groups diverged following surgery, with the H-lesioned rats choosing the large, delayed reinforcer less than sham-operated controls (Figure 10b). Comparison of choice in the last 3 preoperative sessions (Figure 10a) to that in the first 7 postoperative sessions (Figure 10b), using the model lesion_2 _× (pre/post_2 _× delay_5 _× S) revealed a lesion × pre/post interaction (*F*_1,17 _= 6.50, *p *= .021). However, at this point, analysis of postoperative choice patterns on their own (Figure 10b) did not reveal a significant difference between the two groups (lesion: *F*_1,17 _= 3.46, *p *= .08; delay × lesion: *F *< 1, NS); as it did not take account of preoperative choice patterns, this analysis was less powerful. Later, H-lesioned rats diverged further from sham-operated controls and the difference between the two became significant even without taking account of preoperative information (see below). Both groups remained sensitive to the delay postoperatively (sham, effect of delay: *F*_2.1,12.8 _= 4.59,  = .531, *p *= .03; lesion, effect of delay: *F*_1.5,16.5 _= 7.05,  = .374, *p *= .01).

There were no differences between H-lesioned and sham-operated rats in any other measures collected, including the rate of omissions, the latency to initiate trials, the latency to choose a lever, the latency to collect food, and the rate of nosepoking in the food alcove during delays to reinforcement. Data from the 7 baseline postoperative sessions (sessions 20–26) were analysed. Omissions were very infrequent (overall, rats failed to initiate and/or to press a lever on 0.2% of trials) and there were no group differences in the rates of omission (*F *< 1, NS). Initiation latencies did not differ between groups (lesion: *F *< 1, NS; lesion × delay, *F*_2.8,48.0 _= 1.027,  = .706, NS). Neither did choice latencies: an analysis using the model lesion_2 _× (delay_5 _× lever_2 _× S) revealed no significant terms involving lesion (*F*s < 1.06, NS). Food collection latencies were analysed using the model lesion_2 _× (choice_2 _× delay_5 _× S). Predictably, rats were slower to collect the food following choice of the large, delayed reinforcer as the delays got longer (choice × delay: *F*_2.4,38.5 _= 19.8,  = .602, *p *< .001; effect of delay following choice of the small, immediate reinforcer: *F*_2.5,39.7 _= 1.63,  = .62, NS; effect of delay following choice of the large, delayed reinforcer: *F*_2.2,35.8 _= 18.4,  = .559, *p *< .001) but this was not influenced by the lesion (terms involving lesion, maximum *F*_1,16 _= 1.93, NS). The proportion of the delay spent nosepoking did not alter as a function of the delay, and was not affected by the lesion (only applicable to trials on which the large reinforcer was chosen with a non-zero delay; delay, *F*_1.3,22.3 _= 2.38,  = .437, *p *= .131; lesion × delay, *F*_1.3,22.3 _= 1.53,  = .437, NS; lesion: *F *< 1, NS).

### Effects of removing and reintroducing delays to the large reinforcer (experiment 2)

Both H-lesioned and sham-operated rats were sensitive to the removal of delays in alternating sessions, increasing their preference for the large reinforcer during sessions when it was not delayed (Figure 10c). Choice ratios from these sessions were analysed using the model lesion_2 _× (delays/no delays_2 _× trial block_5 _× S). This revealed a delays/no delays × block interaction (*F*_2.5,42.6 _= 15.3,  = .626, *p *< .001). Additionally, there was a main effect of lesion (*F*_1,17 _= 7.23, *p *= .016), indicating a greater overall preference for the smaller reinforcer across these sessions in H-lesioned rats compared to controls, but there were no other significant terms involving lesion (*F*s < 1.05, NS). In sessions when delays were present, both H-lesioned and sham-operated rats showed a within-session shift in preference as the delay increased (sham, effect of delay: *F*_4,24 _= 4.79, *p *= .006; H-lesioned, effect of delay: *F*_2.6,28.1 _= 20.21,  = .638, *p *< .001), and H-lesioned rats chose the smaller, immediate reinforcer more often (lesion: *F*_1,17 _= 5.91, *p *= .026). In sessions when delays were not present, neither group showed a within-session shift in preference (shams: *F*_4,24 _= 2.46, *p *= .073; H-lesioned: *F*_2.1,23.5 _= 1.63,  = .534, NS), though again the H-lesioned rats showed a stronger preference for the smaller reinforcer (*F*_1,17 _= 6.61, *p *= .02).

These analyses suggested that the H-lesioned rats' preference for the larger reward was less than that of shams even when it was not delayed. However, an alternative possibility is that the H-lesioned rats were loath to choose the large reinforcer when it was delayed, and that this generalized to affect preference even when it was not delayed [35,36]. Consequently, subjects were given a further six sessions with no delays present; preference on the last of these sessions is shown in Figure 10d. H-lesioned rats showed a strong preference for the large reinforcer when it was not delayed, just as shams did, with mean choice ratios >94% in all conditions. In this session, there were no group differences (*F*s < 1, NS) and no within-session shift in preference (*F*s < 1, overall and for H-lesioned and sham-operated groups individually).

When delays were reintroduced (sessions 37–42), preference for the larger, delayed reinforcer declined much more sharply in H-lesioned rats than in shams (Figure 10e,f). Preference for the large reinforcer declined first at long delays, then progressively at shorter delays, such that even responding in the zero-delay block was affected. In sessions 37–39, H-lesioned rats chose the large reinforcer less often than shams (lesion: *F*_1,17 _= 5.90, *p *= .027; lesion × delay: *F*_1.9,32.7 _= 1.16,  = .482, NS), with this difference being significant for 10 s and 20 s delays (*p *< .05) but not 0 s (*p *= .075), 40 s (*p *= .058), or 60 s delays (*p *= .054). In sessions 40–42, the pattern was essentially the same (lesion: *F*_1,17 _= 9.47, *p *= .007; lesion × delay: *F*_1.6,28.0 _= 1.286,  = .412, NS), except that individual differences were now significant at all delays (*p *< .05).

### Locomotor activity, body mass, and food consumption

**Figure 11:**
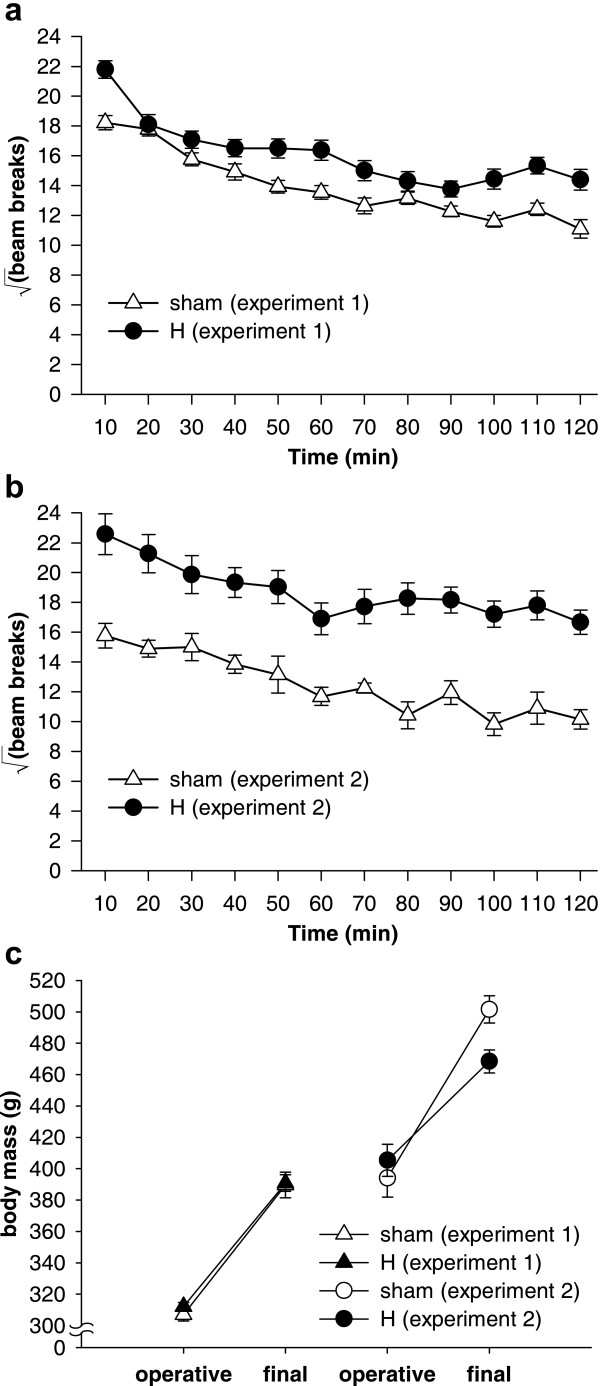
**Locomotor activity in a novel environment and body mass. **Hippocampal-lesioned rats were significantly hyperactive compared to sham-operated controls, in both **(a) **Experiment 1 (*p *= .003) and **(b) **Experiment 2 (*p *< .001). **(c) **Body mass across both experiments. There were no differences between groups preoperatively in either experiment. In Experiment 1, the groups gained weight at the same rate, but in Experiment 2, which lasted longer, the H-lesioned rats weighed less at the end of the experiment (*p *= .01).

H-lesioned animals were hyperactive compared to sham-operated controls in both experiments (Figure 11a,b), as reported previously [37,38]. In Experiment 1, analysis of the square-root-transformed number of infrared beam breaks using the model lesion_2 _× (bin_12 _× S) revealed effects of lesion (*F*_1,41 _= 9.77, *p *= .003), reflecting hyperactivity in the H group, with additional effects of bin (*F*_8.2,335.7 _= 58.4,  = .744, *p *< .001), reflecting habituation, and a lesion × bin interaction (*F*_8.2,335.7 _= 2.95,  = .744, *p *= .003). In Experiment 2, hyperactivity was again observed (lesion: *F*_1,17 _= 24.1, *p *< .001; bin: *F*_8.6,145.7 _= 15.9,  = .779, *p *< .001; lesion × bin: *F *< 1, NS).

In Experiment 1, H-lesioned rats remained the same weight as sham-operated controls throughout, though in Experiment 2, which lasted longer, they gained less weight than shams (Figure 11c). There were no differences between groups preoperatively in either experiment (*F*s ≤ 1.35, NS). In Experiment 1, the groups gained weight at the same rate (lesion × time, *F *< 1, NS; group difference at second time point: *F *< 1, NS). Data from two H-lesioned subjects in Experiment 2 were lost. In Experiment 2, the H-lesioned rats weighed less at the end of the experiment (lesion × time, *F*_1,15 _= 14.5, *p *= .002; group difference at second time point: *F*_1,15 _= 8.56, *p *= .01).

H-lesioned rats consumed their maintenance chow more quickly and consumed more of it, but they did not differ from sham-operated controls in their consumption of the sucrose pellets employed as reinforcers in the behavioural tasks. In 30 minutes, H-lesioned rats consumed more chow (11.2 ± 0.6 g) than shams (8.2 ± 0.8 g) (*F*_1,7 _= 8.36, *p *= .01). However, there were no differences between the mass of sucrose pellets consumed in 30 minutes by H-lesioned rats (17.5 ± 1.2 g) and by shams (18.3 ± 1.6 g) (*F *< 1, NS). H-lesioned rats were quicker to consume 2.5 g of chow (taking 302 ± 18 s) than shams (385 ± 24 s) (Levene's test indicated significant heterogeneity of variance; Mann-Whitney *U*_7,12 _= 18, *p *= .045). However, although H-lesioned rats were also slightly quicker to consume 2.5 g of sucrose pellets (taking 160 ± 9 s) than shams (who took 176 ± 12 s), this difference was not significant (*F*_1,17 _= 1.24, *p *= .281).

## Discussion

Excitotoxic lesions of the dorsal and ventral hippocampus slightly retarded instrumental learning on a continuous reinforcement (fixed-ratio-1; FR-1) schedule in the absence of response-reinforcer delays. However, H-lesioned rats were only impaired when reinforcement was delivered immediately, and not when it was delivered after a delay. H-lesioned rats were less sensitive to the deleterious effects of response-reinforcer delays on learning, to the extent that with long (20 s) response-reinforcer delays, H-lesioned rats showed numerically better discrimination between the active and inactive levers than shams (Figure 7, Figure 9). Despite this delay-dependent facilitation of instrumental conditioning, H-lesioned rats were less able than shams to choose a delayed, large reinforcer in preference to an immediate, small reinforcer (Figure 10). That is, H-lesioned rats exhibited impulsive choice.

### Pavlovian and instrumental conditioning with delayed reinforcement

Free-operant instrumental conditioning, and instrumental discrimination learning, have long been known to be impaired systematically by response-reinforcer (action-outcome) delays [6-9]. This might be for several reasons [12,16,39] because instrumental responding depends on several processes, including knowledge of the action-outcome contingency, a representation of the instrumental incentive value of the outcome, S-R habits, and the influence of Pavlovian CSs that have motivational significance through processes such as conditioned reinforcement and Pavlovian-instrumental transfer [4,17,18]. Action-outcome delays might affect several of these processes. For example, such delays may hinder the subject's ability to perceive the action-outcome contingency, so the subject is unaware that its actions will result in the outcome. Delays might reduce the value of the goal, so the subject is less willing to work for it. Delays might also impair the process by which S-R habits are reinforced; finally, they might affect the degree to which stimuli associated with reinforcement by Pavlovian conditioning are capable of motivating behaviour. In the present experiment, we did not present explicit stimuli associated with either the response or the reinforcer, to minimize the possible contribution of processes such as conditioned reinforcement. Nevertheless, it is largely an open question which processes contributing to instrumental learning and performance are the ones most affected by response-reinforcer delays; for example, it is not present known whether responses acquired with delayed reinforcement are governed by a different balance of habits and goal-directed actions than responses acquired with immediate reinforcement.

However, the environmental context has been clearly demonstrated to influence learning with delays. The effect of contextual factors on learning has been demonstrated using two Pavlovian conditioning paradigms: *delay conditioning *and *trace conditioning*. When a CS is followed by a US and the two overlap and are contiguous in time, the paradigm is known as 'delay' conditioning (the terminology is somewhat confusing). When a CS is followed by a US and the two do *not *overlap, so that there is a gap between the end of the CS and the start of the US, the paradigm is known as 'trace' conditioning (because the US must be associated with a 'trace' of the CS) [39]. 'Trace' Pavlovian conditioning, with a CS-US gap, results in poorer learning than 'delay' Pavlovian conditioning, even if the interstimulus interval (ISI; the time between the onset of the CS and the onset of the US) is held constant [39-48], just as insertion of an action-outcome gap retards instrumental conditioning [7-9]. Several lines of evidence suggest that contextual competition is a cause of reduced responding to the discrete CS in trace conditioning. As the trace gap is lengthened, conditioned responses (CRs) tend to occur to the context instead of to the discrete CS [20,43,49-52]. Trace conditioning can be improved by the addition of a 'filler' stimulus during the CS-US gap, which might decrease contextual competition or act as a secondary or conditioned reinforcer [48,53,54]. The smaller the ratio of the ISI to the intertrial interval (ITI; the time between the end of the US and the start of the next trial's CS), the faster conditioning proceeds [5], and one explanation of this is that long ITIs reduce the strength of context-US associations, making CS-US associations more salient. Finally, pre-exposure to the context in the absence of any US improves subsequent conditioning to a discrete CS, as would be expected under the contextual competition account since pre-exposure should produce latent inhibition of the context (see [55]).

The idea that reinforcing outcomes may be associated with either a discrete predictor (such as a Pavlovian CS or an instrumental response) or the background context, and that the two compete in some way for such association, also explains observations concerning the effect of contextual manipulations on instrumental conditioning [9,17]. Dickinson *et al. *[9] trained rats on a free-operant, FR-1 schedule of reinforcement very similar to the one used in the present experiments. They found that the rate of learning, and the asymptotic level of responding, declined across groups as the response-reinforcer delay was increased from 0 to 32 s; rats trained with a 64-s delay failed to learn at all, compared to yoked controls. However, when rats were exposed to the training context prior to training (in the absence of the lever or any reinforcers) their learning was improved, and successful discrimination was seen even with a delay of 64 s. This is exactly what would be expected if a process of contextual competition was operating. The subject's task is to distinguish *P*(outcome | action) from *P*(outcome | no action), or, making the contribution of the context explicit, to distinguish *P*(outcome | action + context) from *P*(outcome | context). Pre-exposure to the context would be expected to produce latent inhibition to the context, reducing the strength of context-outcome associations. Viewed another way, non-reinforced exposure to the context forces the subjects to experience a zero-response, zero-reinforcer situation, i.e. *P*(outcome | context) = 0. When they are then exposed to the instrumental contingency, such that *P*(outcome | action + context) > 0, this prior experience may enhance their ability to detect the instrumental contingency Δ*P *= *P*(outcome | action) – *P*(outcome | no action). This interpretation is also supported by the demonstration that delivering 'free' rewards (not contingent upon any response of the subject) during the contextual pre-exposure reduces the beneficial effect of this pre-exposure on instrumental learning [9]; by increasing *P*(outcome | context), this reduces the subject's ability to detect the contingency [56-58]. Thus, the formation of context-outcome associations may explain the ability of action-outcome delays to retard instrumental learning.

### Contribution of the hippocampus to instrumental conditioning with immediate reinforcement

In the present study, excitotoxic hippocampal lesions impaired instrumental conditioning on an FR-1 schedule in the absence of response-reinforcer delays. This contrasts with the findings of Corbit *et al. *[59] that electrolytic lesions of the dorsal hippocampus did not affect the acquisition of instrumental responding on a training schedule that progressed from a fixed-interval-20-s (FI-20) schedule up to a random-ratio-20 (RR-20) schedule, consistent with earlier results [60]. Rats with excitotoxic NMDA lesions of the dorsal hippocampus also responded at similar, or greater, rates than sham-operated controls in this training regimen [61]. Obviously, the discrepancy between these findings and the present results might be due to the differences in the schedules used (FR-1 versus RR-20) or in the lesion (dorsal + ventral hippocampus versus dorsal hippocampus only). However, it is less likely that the impairment was due to a primary motivational difference: although the H-lesioned rats gained less mass than shams, the food consumption tests showed that they ate as many of the pellets used as reinforcers as shams, and as quickly, suggesting that the impairment observed was not due to reduced primary motivation for the food. It is, however, possible that it represents a rate-dependent impairment (i.e. that the H-lesioned rats in the zero-delay condition were responding at their maximum possible rate).

Furthermore, electrolytic lesions of the dorsal hippocampus have previously been shown to render rats insensitive to changes in the instrumental action-outcome contingency, but in a very specific manner [59,61]. One way to test subjects' sensitivity to this contingency is to train them to respond on two levers for two different outcomes, and then to deliver one of the outcomes non-contingently, as well as contingent upon the response. Subjects that are sensitive to the action-outcome contingency should selectively reduce their responding for the foodstuff being delivered non-contingently [62]. Electrolytic dorsal hippocampal lesions impaired this ability, though not the ability to discriminate the two foodstuffs or to respond to changes in their value [59,61]. This may have been because the lesion affected contextual conditioning: if an animal cannot associate non-contingent rewards with the context, it may erroneously associate them with its own action. However, excitotoxic lesions of the dorsal hippocampus did not produce this effect, which was reproduced instead by excitotoxic lesions of the entorhinal cortex and subiculum [61]; lesions of these structures have also been shown to impair contextual conditioning in Pavlovian tasks [29,63-67] (though see [24,68]).

### Contribution of the hippocampus to instrumental conditioning with delayed reinforcement

In contrast, when a delay was imposed between responding and reinforcement in an FR-1 schedule, H-lesioned rats were not impaired at instrumental conditioning, and were even somewhat facilitated in learning, relative to shams, when the reinforcer was delayed by 20 s. Since H-lesioned rats were impaired in the absence of delays, this indicates a delay-dependent improvement in learning, relative to shams. Furthermore, asymptotic rates of responding were reduced less by the delay in H-lesioned rats than in controls. The facilitation of learning after a lesion strongly suggests that the lesion has disrupted one process or strategy that normally competes with another process involved in solving the task (e.g. [69,70]). Given the involvement of the hippocampus in contextual conditioning [20-26,28-30,33,34], the most obvious explanation is that the lesions facilitated instrumental conditioning with delayed reinforcement by reducing competition from context-reinforcer associations that normally hinder the formation or expression of response-reinforcer associations.

Certain simple explanations of the present results can be ruled out. The delay-dependent impairment makes an explanation in terms of differences in primary motivation for the food *per se *unlikely, and the use of a control (inactive) lever means that differences in responding were not attributable to differences in general activity levels, but instead to the contingencies in force on the active lever. Our results also indicated that when there were programmed delays to reinforcement, H-lesioned animals experienced shorter response-reinforcer collection delays, partly because they collected the reinforcer more promptly than shams. This effect probably improved learning in the delay conditions. However, in addition to this effect, there was a further delay-dependent improvement exhibited by H-lesioned rats: even allowing for the shorter response-collection delays that they experienced, their instrumental learning was impaired less by delays than that of sham-operated controls.

The role of the hippocampus in learning an instrumental response with delayed reinforcement has been examined before, though in a very different way. Port *et al. *[71] found that aspirative lesions of the dorsal hippocampus did not impair appetitive instrumental conditioning with delayed reinforcement. Numerically, lesioned rats were slightly faster to learn (to reach a criterion number of reinforced responses) than shams, but the difference was not significant. This is certainly consistent with the present results, in which a delay-dependent improvement was seen as a result of excitotoxic lesions of the dorsal and ventral hippocampus. However, direct comparison is difficult. Firstly, the lesion extent was different. Secondly, aspirative lesions destroy not just overlying cortex but also fibres of passage (axons of non-hippocampal neurons traversing the hippocampus) [72]. Thirdly, the task used by Port *et al. *[71] was quite different to that used in the present study: lever presses led to the delivery of responses after a 5-s delay, while responses during the delay were not reinforced; thus, higher rates of responding inevitably reduced the action-outcome contingency. Fourthly, no other delays were tested and no zero-delay condition was used, so any delay-dependent changes would not have been apparent. Fifthly, no control lever was present, so that responding could only be compared across conditions (inferences from a single condition being potentially confounded with general activity levels). Finally, an autoshaping procedure was used to train the rats, and autoshaping is itself known to be impaired by hippocampal lesions [32,73,74], so this may have mitigated against finding an improvement in the lesioned group.

### Contrasting the effects of hippocampal lesions on instrumental and Pavlovian conditioning involving delayed reinforcement

Hippocampal lesions have also been found to affect Pavlovian conditioning involving temporally non-contiguous stimuli. Trace (non-contiguous) conditioning, described above, is clearly analogous to the instrumental conditioning task with delayed reinforcement used in the present study, which had response-reinforcer gaps [39]. Hippocampal lesions have been reported to impair trace conditioning to a discrete explicit CS, sparing delay (contiguous) conditioning [40,41,44,75-78]. Trace discrimination learning can also be impaired by hippocampal lesions [79]. Thus, hippocampal lesions appear to impair the acquisition of a Pavlovian CR when there is a gap between CS and US. Indeed, it has been suggested that the hippocampus is particularly important for associating discontiguous events – that is, when there is a temporal, or indeed spatial, discontiguity or gap between two events to be associated [80]. In the current study, however, hippocampal lesions delay-dependently facilitated (relative to shams) the acquisition of an instrumental response when there was a gap between action and outcome. What accounts for these apparently contradictory results?

Firstly, the neural differences between trace and delay conditioning may be not as great as it first seems. The fact that hippocampal lesions have often been shown to impair trace conditioning, but not delay conditioning, may be because trace conditioning is more difficult. If delay conditioning is rendered as hard as trace conditioning by extending the delay ('long-delay conditioning'), the hippocampus is required [44]; similarly, H-lesioned subjects can exhibit trace conditioning if pretrained in a delay conditioning paradigm [44,77], some trace discrimination tasks are intact after hippocampal lesions [81], and the hippocampus is not required for expression of the trace response after learning [82].

Secondly, it may be that a representation of context can help trace (and perhaps long-delay) Pavlovian conditioning, while contextual associations only hinder instrumental responding in the present task, so a lesion that disrupts contextual processing has a differential effect on the two. For example, if the CS during trace conditioning acts as an occasion-setter, signalling that the ensuing context will be followed by a US (an example of feature-positive discrimination), then hippocampal lesions, which can impair both feature-positive discrimination [83] and contextual conditioning, might be expected to impair trace conditioning more than delay conditioning (N.J. Mackintosh, personal communication, 12 October 2004). Yet in instrumental conditioning with delayed reinforcement, as examined here, context-outcome associations can only hinder the learning of response-outcome associations, so hippocampal lesions might be expected to improve learning in a delay-dependent fashion, as was observed. A study by Desmedt *et al. *[43] supports the hypothesis that trace and delay conditioning endow the context with qualitatively different roles and that the hippocampus contributes differentially to these roles (though they suggest instead that the context is associated directly with the US in 'trace' conditioning, and that the context and discrete CS act as occasion-setters for each other in some way in 'delay' conditioning [33], with hippocampal lesions facilitating this occasion-setting and impairing direct context processing). Thus, although contexts clearly have several associative functions, the contribution to the hippocampus to these processes is by no means clear cut [33,83,84].

### Effect of hippocampal lesions on choice involving delayed reinforcement

If lesions of the hippocampus reduce the normal deleterious effects of delays on the ability to associate actions with their outcomes, it might be expected that they would also improve subjects' ability to choose a delayed, large reward in preference to an immediate, small reward. Instead, the opposite pattern of results was observed: postoperatively, H-lesioned rats made impulsive choices, preferring the immediate, small reward. This preference was flexible, responding to changes in the contingencies within the task, and all subjects readily reverted to choosing the large reward when all delays were removed, indicating that they could discriminate the large and small rewards and continued to prefer the large reward when it was not delayed. Upon reintroduction of the delays, however, the preference for the large reward collapsed in the H-lesioned group much more prominently than in shams, indicating that they were less tolerant of delays to reinforcement.

The present results are also somewhat similar to those reported by Rawlins *et al. *[85], who examined choice between certain and uncertain rewards. Normal rats preferred immediate certain reward to immediate uncertain reward, and also preferred delayed certain reward to immediate uncertain reward; however, rats with hippocampal or medial septal lesions were less tolerant of the delay (or more tolerant of the uncertainty), preferring immediate uncertain reward to delayed certain reward.

Some simple explanations for this effect may readily be ruled out. It is unlikely that lesioned rats' impulsive choice was caused by lower motivation to obtain the food, for two reasons. First, there were no differences in the rate at which they consumed the sucrose pellets used as the reinforcer. Second, the performance of H-lesioned rats was not similar in other respects to that of a subject with lower primary motivation, such as a sated rat [86]; for example, they did not make more omissions than sham-operated controls. It is also unlikely that H-lesioned rats' preference for the small, immediate reward were the consequence of a positional bias away from the lever producing large, delayed reward: when the delays were omitted, all the H-lesioned rats readily and consistently chose the large reinforcer, only to prefer the small reinforcer again when delays were re-introduced. Furthermore, it is difficult to see that impaired contextual conditioning could explain the pattern of results; as discussed above, the absence of context-reinforcer associations should help, rather than hinder, the ability to associate actions with delayed outcomes. Likewise, although context-response associations may influence instrumental responding and contexts may act as occasion-setters (signalling the operation of a particular action-outcome contingency), there is no *a priori *reason to believe that the context should differentially cue instrumental responding more when the outcome is delayed; neither is there conclusive evidence suggesting that hippocampal lesions impair such a process (see [33]).

One obvious difference between the two experiments is that in Experiment 1, lesions were made before training, whereas in Experiment 2, lesions were made after training. Hence it is possible that hippocampal lesions selectively impair the *retrieval *of a well-learned instrumental response or action-outcome contingency involving delayed outcomes, while sparing those involving immediate outcomes. However, there are two reasons why this scenario is unlikely. Firstly, animals must be able to perform an action in order for that action to be conditioned instrumentally. Experiment 1 demonstrated that H-lesioned rats were able to acquire instrumental responses for delayed reinforcement at least as well as, if not better than, sham-operated controls. Therefore, it is unlikely that hippocampal lesions selectively impair the *performance *of instrumental responses for delayed outcomes. Secondly, this idea would not explain why H-lesioned rats showed a reduced preference for the large, delayed reinforcer upon reintroduction of delays, *after *they had shown a strong postoperative preference for the large reinforcer when delays had been consistently omitted. This required new learning on their part, and since Experiment 1 demonstrated that learning with delayed reinforcement is normal in H-lesioned rats, this would predict self-controlled rather than impulsive choice upon reintroduction of delays.

The task used does not determine whether H-lesioned rats exhibit altered sensitivity to reinforcer *magnitude *or *delay*, for either abnormality might produce impulsive choice [87]. Although H-lesioned rats were able to discriminate in absolute terms between the large and the small reinforcer (consistent with previous studies, e.g. [88]), it is possible that they discriminated between the reinforcer magnitudes to a lesser extent than shams. In this scenario, H-lesioned rats might have exhibited impulsive choice simply because the perceived value of the large reinforcer was not subjectively big enough to compensate for the normal effects of the delay. Alternatively, H-lesioned rats may perceive reward magnitudes normally, and exhibit impulsive choice because they are specifically hypersensitive to (intolerant of) the effects of delays to reinforcement. Such evidence as exists suggests that H-lesioned rats perceive reward magnitude normally [88,89]. Experiment 1 indicated that H-lesioned rats are somewhat better than shams at instrumental conditioning with action-outcome delays of >10 s. This suggests that H-lesioned rats associated the action with the delayed outcome normally in Experiment 2, so if they also perceived its magnitude normally, then it is likely that they valued the delayed outcome less.

The present results may also be explained in terms of altered temporal perception. For example, a lesion that increased the speed of an 'internal clock' [90] might affect choice prospectively in this task (i.e. the lesioned subject perceives itself to be at a later time-point in the session than it actually is, hastening the within-session shift towards the immediate lever), or might affect retrospective choice (i.e. the lesioned subject experiences the delay to the large reinforcer as longer than it actually is, causing it to value the reinforcer less than shams). The evidence for the role of the hippocampus in temporal perception is inconclusive: some studies have found that aspirative hippocampal lesions did not affect timing behaviour [91-94], whereas others have suggested that lesions of the hippocampus or fimbria/fornix speed up an internal clock, or reduce the estimation of time periods when a stimulus being timed is interrupted [80,95-98].

Finally, the hippocampus is heavily connected to a number of other structures known to play a role in subjects' relative preference between immediate, small and delayed, large rewards. Lesions of the nucleus accumbens core (AcbC), basolateral amygdala, and orbitofrontal cortex (OFC) have all been found to produce impulsive choice [35,99-101]. OFC lesions appear to alter the processing of reward magnitude as well as delay, and lesions here have produced both impulsive and self-controlled choice under different circumstances [99-102]. AcbC lesions appear to have a more selective effect on the processing of delays, impairing both preference for, and learning with, delayed rewards in the absence of effects on reward magnitude processing [35,103]. Although the hippocampal formation projects heavily to most of the nucleus accumbens (via the subiculum) [104] and H-lesioned rats in the present study showed the impulsive choice known to be exhibited by AcbC-lesioned rats, H-lesioned rats showed the opposite effect to AcbC-lesioned rats in the simple instrumental learning task, being delay-dependently improved rather than impaired relative to shams.

## Conclusion

We have demonstrated that excitotoxic lesions of the hippocampus ameliorate the deleterious effects of response-reinforcer delays on instrumental learning. Hippocampal-lesioned rats responded slightly less than controls in the absence of delays, but they became better at learning (relative to shams) as the delays increased, in a delay-dependent fashion. This may have been because the lesion hindered the formation of context-outcome associations, promoting response-outcome association instead. In contrast, lesioned rats exhibited impulsive choice, preferring an immediate, small reward to a delayed, larger reward, even though they preferred the large reward when it was not delayed. Thus, lesioned rats were better at learning with delayed reinforcement but worse at choosing it, suggesting that self-controlled choice and learning with delayed reinforcement tax different psychological processes.

## Methods

### Overview of experiments

#### Experiment 1: Effects of hippocampal lesions on acquisition of instrumental responding with delayed reinforcement

Forty-eight rats received excitotoxic lesions of the hippocampus (*n *= 32) or sham lesions (*n *= 16). Five died postoperatively. Subject were next trained in a task in which they had continuous access to two identical levers; one lever delivered a single food pellet each time it was pressed, and the other lever had no effect. For some rats, the food pellet was delivered immediately after the lever press (0 s condition; 9 H-lesioned rats and 5 shams). For others, each pellet was delayed by either 10 s (9 H, 6 sham) or 20 s (9 H, 5 sham). Subjects were trained for 14 sessions. They then had their locomotor activity assessed, and finally they were killed and perfused for histology.

#### Experiment 2: Effects of hippocampal lesions on choice involving delayed reinforcement

Twenty-four naïve rats were first trained to press levers for food and to nosepoke to initiate lever presentations in discrete trials. Subjects were then trained on a choice-of-delayed-reinforcement task (described below) for 19 sessions. After this, they were assigned to matched groups (as described below) to receive lesions of the hippocampus (H, *n *= 16) or sham lesions (sham, *n *= 8). Following recovery, they were retested on the basic task for 7 sessions to obtain a baseline measure of performance. After this, 4 sessions were given in which all delays were omitted in alternate sessions (DNDN design; D = delays present, N = no delays). Half of the subjects began this test with the delays present, and half with no delays (counterbalanced across groups). As a deficit was observed during testing (before histological data were available), further behavioural tests were given to elucidate the nature of the deficit. All subjects were given a further 6 sessions with no delays, in an attempt to re-equalize the two groups' performance and ensure that all animals would come to prefer the lever producing the large reinforcer. Delays were then re-introduced for a further 6 sessions. All subjects then underwent a food consumption test and had their locomotor activity assessed; finally, they were killed and perfused for histology.

### Subjects and housing conditions

Subjects were male Lister hooded rats (Harlan-Olac UK Ltd) housed in a temperature-controlled room (minimum 22°C) under a 12:12 h reversed light-dark cycle (lights off 07:30 to 19:30). Subjects were approximately 15 weeks old on arrival at the laboratory and were given a minimum of a week to acclimatize, with free access to food, before experiments began. Experiments took place between 09:00 and 21:00, with individual subjects being tested at a consistent time of day. Subjects had free access to water, and were housed either in groups of four (Experiment 1) or in pairs (Experiment 2). During behavioural testing, they were maintained at 85–90% of their free-feeding mass using a restricted feeding regimen. Feeding occurred in the home cages at the end of the experimental day. All procedures were subject to UK Home Office approval (Project Licence 80/1767) under the Animals (Scientific Procedures) Act 1986.

### Excitotoxic lesions of the hippocampus

Subjects were anaesthetized with Avertin (2% w/v 2,2,2-tribromoethanol, 1% w/v 2-methylbutan-2-ol, and 8% v/v ethanol in phosphate-buffered saline, sterilized by filtration, 10 ml/kg intraperitoneally) and placed in a Kopf or Stoelting stereotaxic frame (David Kopf Instruments, Tujunga, California, USA; Stoelting Co., Wood Dale, Illinois, USA) fitted with atraumatic ear bars. The skull was exposed and a dental drill was used to remove the bone directly above the injection and cannulation sites. The dura mater was broken with the tip of a hypodermic needle, avoiding damage to underlying venous sinuses. Excitotoxic hippocampal lesions targeted both the dorsal hippocampus and the ventral hippocampus. Lesions were made by injecting 0.09 M *N*-methyl-D-aspartic acid (NMDA; Sigma, UK) [72] through a glass micropipette (tip diameter 50–100 μm), using the coordinates, volumes, and timings shown in Table 1. The toxin had been dissolved in 0.1 M phosphate buffer (composition 0.07 M Na_2_HPO_4_, 0.028 M NaH_2_PO_4 _in double-distilled water, sterilized by filtration) and adjusted with NaOH to a final pH of 7.2–7.4. Sham lesions were made in the same manner except that vehicle was infused. At the end of the operation, animals were given 15 ml/kg of sterile 5% w/v glucose, 0.9% w/v sodium chloride intraperitoneally. Lesioned animals were given 0.2 ml of 5 mg/ml diazepam (Roche Products Ltd, UK) i.m. to prevent seizures. They were given two weeks to recover, with free access to food, and were handled regularly. Any instances of postoperative constipation were treated with liquid paraffin orally and rectally. At the end of this period, food restriction commenced or was resumed.

**Table 1:** Lesion coordinates. Excitotoxic lesions of the entire hippocampus were made by injecting 0.09 M NMDA at the coordinates shown (see Methods). Along the anteroposterior (AP), mediolateral (ML), and dorsoventral (DV) axes, positive coordinates are in the anterior, left, and superior directions respectively. All coordinates are in mm. DV coordinates are measured from the dura above the injection site.

Region within hippocampus	Sites per hemisphere	AP	ML	DV	Volume injected per site	Duration of each infusion	Time allowed for diffusion after each infusion
Dorsal	2	-2.8	± 1.6	-3.3	0.4 μl	4 min	3 min
		-4.2	± 2.6	-3.0	0.4 μl	4 min	3 min
Ventral	4	-4.8	± 4.8	-6.0	0.2 μl	2 min	3 min
		-5.3	± 4.6	-4.2	0.2 μl	2 min	3 min
		-5.3	± 4.6	-6.0	0.2 μl	2 min	3 min
		-5.8	± 4.6	-4.2	0.2 μl	2 min	3 min

### Behavioural apparatus

Behavioural testing was conducted in one of two types of operant chamber of identical configuration (from Med Associates Inc., Georgia, Vermont, USA, or Paul Fray Ltd, Cambridge, UK). Each chamber was fitted with a 2.8 W overhead house light and two retractable levers on either side of an alcove fitted with an infrared photodiode to detect head entry and a 2.8 W lightbulb ('traylight'). Sucrose pellets (45 mg, Rodent Diet Formula P, Noyes, Lancaster, New Hampshire, USA) could be delivered into the alcove. The chambers were enclosed within sound-attenuating boxes fitted with fans to provide air circulation. The apparatus was controlled by software written by RNC in C++ [105] using the Whisker control system [106].

### Instrumental conditioning with delayed reinforcement (experiment 1)

A variety of free-operant schedules may be used to assess instrumental acquisition with delayed reinforcement [9]. We used the simplest possible free-operant schedule [103]: each response scheduled a reinforcer after the programmed delay (Figure 1). In such a schedule, if the subject responds during the delay, the experienced response-reinforcer delay will not match the programmed delay (as the second response is temporally close to the first reinforcer). However, this schedule has the advantage that the response-reinforcer contingency is constant (every response does in fact cause the delivery of reinforcement) and the reinforcement rate is not constrained [9]. So that responding could be attributed to the instrumental response-reinforcer contingency, rather than the effects of general activity or reinforcement itself, responding on the active lever was compared to responding on a control lever that had no programmed consequence. Different groups of lesioned and sham-operated subjects were trained using different delays; the delay was consistent for every subject. Delays of 0, 10, and 20 s were used.

Immediately after subjects were placed in the operant chamber, the sessions began. The houselight was illuminated, and remained on for each 30-min session. Two levers were extended into the chamber. All lever responses were first 'debounced' to 10 ms (i.e. if a response occurred within 10 ms of a previous valid response it was attributed to mechanical bounce and ignored). Other than this, all lever presses and nosepokes into the food alcove were recorded. Responding on the left (active) lever caused a single pellet to be delivered following a delay, under a fixed-ratio-1 (FR-1) schedule (Figure 1). To attribute acquisition of a lever-press response to the instrumental contingency, it is also necessary to control for the effects of reinforcer delivery itself [9]; therefore, responding on the active lever was compared to responding on the right (inactive) lever, which had no programmed consequence. To minimize any potential contribution of conditioned reinforcement to the task, no explicit signals were associated with pellet delivery other than the noise of the pellet dispenser apparatus.

### Lever and nosepoke training prior to the delayed reinforcement choice task (experiment 2)

Subjects were first trained under an FR-1 schedule (where every lever press leads to the immediate delivery of a pellet) with only one lever present, to a criterion of a total of 50 presses on that lever across 30-min sessions, first for the left lever and then for the right. Subjects were then trained on a simplified version of the full task. The session began with the levers retracted and the operant chamber in darkness. Trials began every 40 s with the illumination of the houselight and the traylight. The subject was required to make a nosepoke response within 10 s, or the trial was aborted and the chamber returned to darkness (scored as an omission). If the subject nosepoked within the time limit, the traylight was extinguished and a single lever was presented (left/right at random). Subjects were required to respond on the lever within 10 s or the lever was retracted and the chamber darkened (scored as an omission). Upon pressing the lever, the houselight was switched off, a single pellet was delivered immediately and the traylight was illuminated until either the pellet was collected or 10 s had elapsed, whereupon the chamber was darkened, and the trial was counted as successful. Rats were trained to a criterion of 60 successful trials in one hour (the maximum possible with trials lasting 40 s being 90).

### Choice between small, immediate and large, delayed rewards (experiment 2)

The task was based on Evenden & Ryan's [107] procedure and has been described before [35,86,99]. The session began in darkness with the levers retracted; this was designated the intertrial state. Trials began at 100-s intervals; the format of a single trial is shown in Figure 2. Each trial began with the illumination of the houselight and the traylight. The rat was required to make a nosepoke response, ensuring that it was centrally located at the start of the trial (latency to poke was designated the initiation latency). If the rat did not respond within 10 s of the start of the trial, the operant chamber was reset to the intertrial state until the next trial began and the trial was scored as an omission. If the rat was already nosepoking when the trial began, the next stage followed immediately. Upon a successful nosepoke, the traylight was extinguished and one or both levers were extended. One lever was designated the Delayed lever, the other the Immediate lever (counterbalanced left/right). The latency to choose a lever was recorded. (If the rat did not respond within 10 s of lever presentation, the chamber was reset to the intertrial state until the next trial and the trial was scored as an omission.) When a lever was chosen, both levers were retracted and the houselight was switched off. Choice of the Immediate lever caused the immediate delivery of one pellet; choice of the Delayed lever caused the delivery of 4 pellets following a delay. When reinforcement was delivered, the traylight was switched on. Multiple pellets were delivered 0.5 s apart. If the rat collected the pellets before the next trial began, then the traylight was switched off and time from delivery of the first pellet until a nosepoke occurred was recorded as the collection latency. If the rat did not collect the food within 10 s of its delivery, the operant chamber entered the intertrial state, though collection latencies were still recorded up to the start of the next trial. The chamber was then in the intertrial state and remained so until the next trial. There was no mechanism to remove uneaten pellets, but failure to collect the reward was an extremely rare event.

The delay was varied systematically across the session. A session consisted of 5 blocks, each comprising two trials on which only one lever was presented (one trial for each lever, in randomized order) followed by ten free-choice trials. Preferences were calculated for each block from only those trials on which the subject responded. Delays for each block were 0, 10, 20, 40 and 60 s respectively. As trials began every 100 s, the total session length was 100 minutes; subjects received one session per day.

Preoperatively, subjects were trained on this task for 19 sessions. To allocate subjects into matched groups for surgery, their degree of sensitivity to the effects of delays within each session was assessed for the last three preoperative sessions, by calculating the slope of the linear regression of percentage choice of the large delayed reinforcer against delay for each subject. Rats were ranked by this measure, and rats with equivalent levels of performance were randomized to receive sham or H lesion surgery: the ranked list was divided into ordered triplets, and from each triplet one subject was assigned to the sham group and the other two to the H group, at random.

### Locomotor activity in a novel environment

Locomotor activity was measured in wire mesh cages, 25 (W) × 40 (D) × 18 (H) cm, equipped with water bottles and two horizontal infrared photocell beams situated 1 cm from the floor that enabled movements along the long axis of the cage to be registered. Subjects were placed in these cages, which were initially unfamiliar to them, and their activity was recorded for 2 h. All animals were tested in the food-deprived state.

### Food consumption tests

Food consumption was assessed using four tests, conducted in subjects' home cages (always with only one rat present) on separate days under conditions of food deprivation. (1) Subjects were given free access to the 45-mg sucrose pellets used as reinforcers (Rodent Diet Formula P, Noyes, Lancaster, NH) for 30 minutes; the amount eaten was recorded. (2) This test was repeated with the chow used as the maintenance diet. (3) The time taken to consume 50 sucrose pellets was recorded. (4) The time taken to consume an equivalent mass of chow (2.25 g) was recorded.

### Histology

Rats were deeply anaesthetized with pentobarbitone sodium (200 mg/ml, minimum of 1.5 ml i.p.) and perfused transcardially with 0.01 M phosphate-buffered saline (PBS) followed by 4% paraformaldehyde in PBS. Their brains were removed and postfixed in paraformaldehyde before being dehydrated in 20% sucrose for cryoprotection. The brains were sectioned coronally at 60 μm thickness on a freezing microtome and every third section mounted on chromium potassium sulphate/gelatin-coated glass microscope slides and allowed to dry. Sections were passed through a series of ethanol solutions of descending concentration (3 minutes in each of 100%, 95%, and 70% v/v ethanol in water) and stained for ~5 min with cresyl violet. This stain comprises 0.05% w/v aqueous cresyl violet (Raymond A. Lamb Ltd, Eastbourne, UK), 2 mM acetic acid, and 5 mM formic acid in water. Following staining, sections were rinsed in water and 70% ethanol before being differentiated in 95% ethanol. Finally, they were dehydrated and delipidated in 100% ethanol and Histoclear (National Diagnostics, UK) before being cover-slipped using DePeX mounting medium (BDH, UK) and allowed to dry. The sections were used to verify lesion placement and assess the extent of lesion-induced neuronal loss. Lesions were detectable as the absence of visible neurons (cell bodies of the order of 100 μm in diameter with a characteristic shape), often associated with a degree of tissue collapse (sometimes with consequent ventricular expansion when the lesion was adjacent to a ventricle) and gliosis (visible as the presence of smaller, densely-staining cells).

### Data analysis

Data collected by the chamber control programs were imported into a relational database (Microsoft Access 97) for case selection and analysed with SPSS 11. Figures were created with SigmaPlot 2001/v7 and Adobe Illustrator 8. All graphs show group means and error bars are ± 1 standard error of the mean (SEM) unless otherwise stated. Count data (lever presses and locomotor activity counts), for which variance increases with the mean, were subjected to a square-root transformation prior to any analysis [108]. Homogeneity of variance was verified using Levene's test [109]. General linear models are described as *dependent variable = A*_2 _× *B*_*cov *_× (*C*_5 _× *D*_*cov *_× *S*) where A is a between-subjects factor with two levels, B is a between-subjects covariate, C is a within-subjects factor with five levels, and D is a within-subjects covariate; S denotes subjects in designs involving within-subjects factors [110]. For repeated measures analyses, Mauchly's test of sphericity of the covariance matrix was applied [111] and the degrees of freedom corrected to more conservative values using the Huynh-Feldt epsilon  for any terms involving factors in which the sphericity assumption was violated [112].

## List of abbreviations used

 , Huynh-Feldt epsilon

AcbC, nucleus accumbens core

ANCOVA, analysis of covariance

ANOVA, analysis of variance

CA, cornu ammonis (Ammon's horn)

CR, conditioned response

CS, conditioned stimulus

FI, fixed interval

FR, fixed ratio

H, hippocampus

h, hour

i.m., intramuscular

i.p., intraperitoneal

ISI, interstimulus interval

ITI, intertrial interval

min, minute

NMDA, *N*-methyl-D-aspartate

OFC, orbitofrontal cortex

*P*(A | B), probability of A occurring, given that B has occurred

*P*(A), probability of event A occurring

PBS, phosphate-buffered saline

RR, random ratio

SED, standard error of the difference between means

SEM, standard error of the mean

S-R, stimulus-response

US, unconditioned stimulus

v/v, volume per unit volume

w/v, weight per unit volume

## Authors' contributions

RNC conceived and designed the studies, supervised THCC, and wrote the software. THCC participated in the design of the studies, performed the surgery, tested the animals, and processed the histological material. The work contributed to THCC's M.Phil. thesis. Both authors analysed the results and wrote the manuscript.
